# Reactive Carbonyl Species *In Vivo*: Generation and Dual Biological Effects

**DOI:** 10.1155/2014/417842

**Published:** 2014-01-21

**Authors:** Halyna M. Semchyshyn

**Affiliations:** Department of Biochemistry and Biotechnology, Vassyl Stefanyk Precarpathian National University, 57 Shevchenko Street, Ivano-Frankivsk 76025, Ukraine

## Abstract

Reactive carbonyls are widespread species in living organisms and mainly known for their damaging effects. The most abundant reactive carbonyl species (RCS) are derived from oxidation of carbohydrates, lipids, and amino acids. Chemical modification of proteins, nucleic acids, and aminophospholipids by RCS results in cytotoxicity and mutagenicity. In addition to their direct toxicity, modification of biomolecules by RCS gives rise to a multitude of adducts and cross links that are increasingly implicated in aging and pathology of a wide range of human diseases. Understanding of the relationship between metabolism of RCS and the development of pathological disorders and diseases may help to develop effective approaches to prevent a number of disorders and diseases. On the other hand, constant persistence of RCS in cells suggests that they perform some useful role in living organisms. The most beneficial effects of RCS are their establishment as regulators of cell signal transduction and gene expression. Since RCS can modulate different biological processes, new tools are required to decipher the precise mechanisms underlying dual effects of RCS.

## 1. Introduction

Reactive carbonyl species (RCS) include a large number of biological compounds with one or more carbonyl groups that are continuously produced in various groups of organisms, from bacteria to man, and mainly known for their damaging effects. The steady-state concentration of RCS is maintained in a certain range and, according to homeostasis theory, fluctuates in the cell similar to other parameters. However, RCS level may leave the range due to changes occurring in RCS production and/or elimination. An increase in steady-state level of reactive carbonyls is the key cause of the phenomenon called carbonyl stress, a contributing factor to aging, pathogenesis of metabolic syndrome, chronic complications associated with diabetes and renal failure, neurodegenerative, and other disorders [1–9]. On the other hand, constant persistence of RCS in the cells at low concentrations can be considered to be the emergence of RCS as an important part of immune response, regulators of gene expression, and cellular signaling messengers [2, 8, 10]. Therefore, like other reactive species, RCS play a dual role *in vivo* which appears to be dose- and time-dependent [10–15].

## 2. Generation of Reactive Carbonyls *In Vivo*

**Figure 1:**
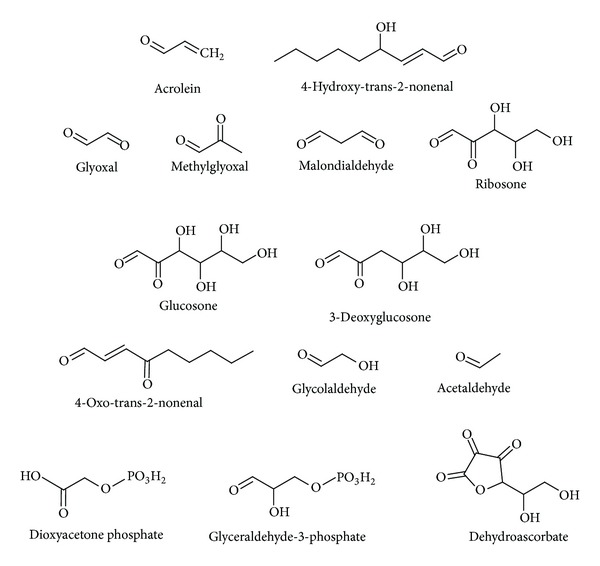
The structures of most common biological reactive carbonyl species.

Reactive carbonyls are compounds found widespread throughout biological life and can be endogenous or exogenously derived. More than 20 RCS have been identified in biological samples [10].  Figure 1 demonstrates most common saturated and unsaturated RCS detected in living organisms. Some reactive carbonyls (e.g., acrolein, crotonaldehyde, glyoxal, acetone, and formaldehyde) are ubiquitous industrial pollutants which can readily enter the cell from the environment [16–18]. Other exogenous sources of RCS are products of organic pharmaceutical chemistry, cigarette smoke, food additives, and browned food [19–24].

There is increasing evidence that RCS are produced endogenously [10, 15, 25, 26].  Table 1 demonstrates most widespread biological reactive carbonyls generated during nonenzymatic or enzymatic reactions* in vivo*. A wide diversity of intracellular unstable RCS is readily produced by such nonenzymatic processes as lipid peroxidation, amino acid oxidation, and glycation [2, 9, 10, 27–31].

A key feature of lipid peroxidation is the free radical chain breakdown of polyunsaturated fatty acid residues in cholesterol esters, phospholipids, and triglycerides that yields a broad array of RCS, including malondialdehyde (MDA), glyoxal (GO), 4-hydroxy-2-nonenal (4-HNE), and 4-oxo-trans-2-nonenal [2, 10, 32, 33]. Such amino acids as threonine and glycine can be converted to RCS (e.g., methylglyoxal (MGO)) or their precursors (e.g., aminoacetone and succinylacetone) during oxidative modification [34].

Glycation, a nonenzymatic process involved reducing carbohydrates (e.g., glucose and fructose), attracts considerable attention during the last decades [15, 35–37]. This could be attributed to either an excessive consumption of carbohydrate sweeteners in the modern human diet [37] or their opposite dual effects *in vivo* [11, 14, 38–45]. Potential mechanisms underlying both detrimental and beneficial effects of reducing carbohydrates are under debate. Recently we suggested the involvement of RCS and reactive oxygen species (ROS) in both the cytotoxic and defensive effects of such reducing carbohydrate as fructose [14, 15].

**Figure 2:**
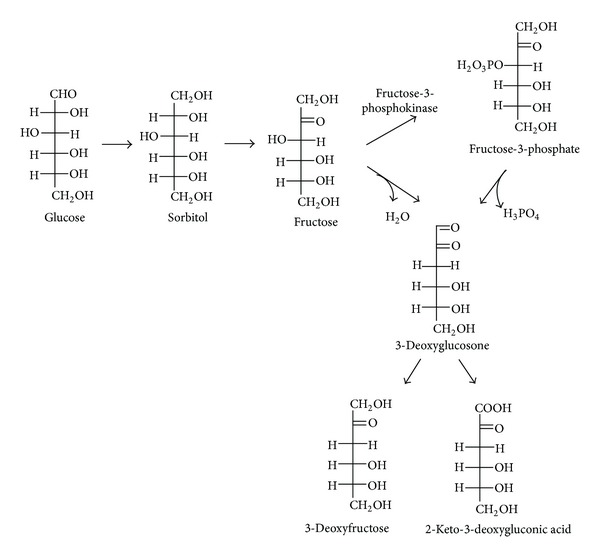
Formation of methylglyoxal as a by product of glycolysis.

**Figure 3:**
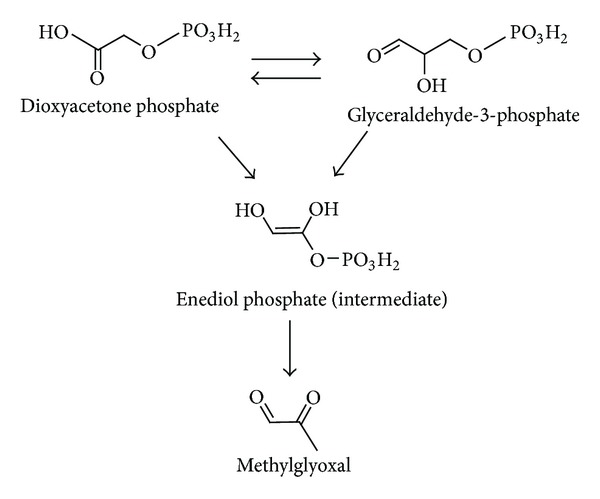
Polyol pathway as a source of formation of reactive carbonyl species.

In several enzymatic pathways involving carbohydrates MGO, GO, and 3-deoxyglucosone (3-DG) are generated as side products (Table 1). The polyol pathway is a two-step metabolic pathway in which glucose is reduced to sorbitol, which is then converted to fructose (Figure 2). Generally, polyol pathway is associated with the production of 3-DG [46, 47]. Glycolysis is probably the most thoroughly studied metabolic pathway, the major enzymatic source of MGO* in vivo* [48–51].  Figure 3 demonstrates the mechanisms of MGO generation in glycolysis. Enediol phosphate, an intermediate of triosophosphate isomerase reaction, may escape from the active site of the enzyme and be rapidly decomposed to MGO and inorganic phosphate. MGO can also be formed from the intermediates in the enzymatic oxidation of ketone bodies [29, 31]. Different RCS are generated *in vivo* by activated human phagocytes. It has been found that stimulated neutrophils employed the myeloperoxidase-H_2_O_2_-chloride system produce *α*-hydroxy- and *α*,*β*-unsaturated aldehydes from hydroxy-amino acids in high yield [52].

The steady-state concentration of such carbonyl metabolic intermediates as acetaldehyde, glyceraldehyde-3-phosphate, and dioxyacetone phosphate are typically low in the cell because of their rapid utilization by the next step of the pathway. However, the concentration of reactive carbonyl by products in enzymatic reactions is not so tightly controlled *in vivo*. Therefore, under certain conditions, biological effects of these carbonyl side products may be more potent than the effect caused by carbonyl metabolic intermediates.

## 3. Deleterious Effects of Reactive Carbonyls

Like most intermediates and by products of metabolism, RCS are electrophilic and therefore highly reactive toward different cellular constituents majority of which are nucleophiles [32]. It should be noted that unsaturated RCS are usually an order of magnitude more reactive than their saturated counterparts. Therefore, most of biological damages caused by RCS are related to *α*, *β*-unsaturated aldehydes, dialdehydes and keto-aldehydes [2, 53]. Such strong nucleophilic sites as thiol, imidazole, and hydroxyl groups of biomolecules are the most attractive targets for electrophilic attacks. MDA, GO, MGO, 3-DG, glucosone, and ribosone are highly reactive *α*- or *β*-dicarbonyl compounds (Figure 1). Dicarbonyls react with nucleophilic groups of macromolecules like proteins, nucleic acids, and aminophospholipids, resulting in their irreversible modification and formation of a variety of adducts and cross links collectively named advanced glycation or lipoxidation end products (AGEs, ALEs) [53–59].

**Figure 4:**
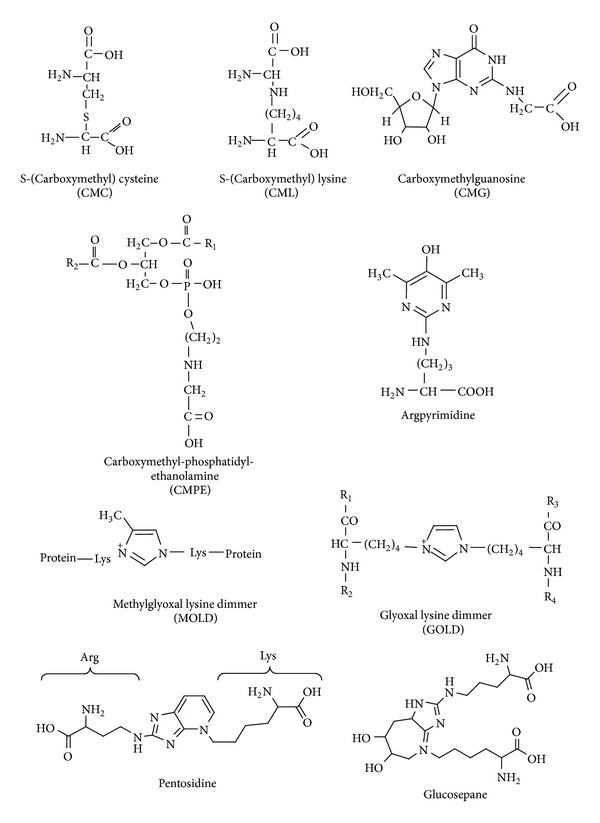
The structures of most common biological advanced lipoxidation and glycation end products.

In general, ALEs and AGEs are poorly degraded complexes, accumulation of which increases with age. These adducts have been detected in various tissues and peripheral blood and considered to be pathogenic. Carboxymethyl phosphatidylethanolamine and carboxymethylguanosine represent the ALEs/AGEs derived from GO and MGO interaction with nucleic acids and phospholipids, respectively (Figure 4). Adducts such as GO-lysine dimmer, MGO-lysine dimmer, carboxymethyllysine, carboxymethylcysteine, and argpyrimidine are the most common ALEs/AGEs resulted from protein modification (Figure 4). RCS react preferentially with arginine, cysteine, and lysine residues with high reaction rates [35]. Physiological RCS may play important role in pathogenesis because of high abundance of the amino acid residues within protein active sites [60–63]. Carboxymethyllysine was the first AGE isolated from glycated proteins *in vivo* and together with pentosidine and glucosepane (Figure 4) was later recognized as one of the most important indicator of glycation in living organisms [55, 57, 64]. RCS as well as ALEs/AGEs are found to induce most features of the metabolic syndrome, including glucose intolerance and hyperglycemia, abdominal obesity, elevated blood pressure, inflammation, and renal injury [57, 59]. It should be noted that ALEs/AGEs may continue covalent interactions with biomolecules giving more complex cross-links. In addition, ALEs and AGEs are efficient sources of RCS and ROS *in vivo* [1, 28, 58, 65–67].

Generally, biological effects by RCS seems somewhat similar to those by ROS thus it can be expected that physicochemical properties of both reactive groups should be similar as well. However, RCS have a relatively long half-life time and higher stability, in contrast to ROS. For instance, reactive carbonyls have average half-life from minutes to hours [2, 53]. At the same time, half-life of some ROS ranges from 10^−9^ to 10^−6^ s [68, 69]. It is well known that such uncharged ROS as H_2_O_2_ and HO_2_^•^ are able to cross biological membranes and diffuse for relatively long distances in the intracellular environment. Higher stability of uncharged RCS allows them even to escape from the cell and interact with targets far from the sites of their generation.

## 4. Beneficial Impacts of Reactive Carbonyl Species

Although excessive RCS may lead to pathological disorders and accelerate aging, the reactive species may also exert beneficial effects at low levels. An obvious question arises: what are the “excessive” and “low” concentrations of RCS in the cell? A measurement of physiological concentration of RCS is often problematic due to (i) a vast variety of RCS generated *in vivo* by different mechanisms; (ii) simultaneous production, degradation, and excretion of RCS; (iii) dependence of the above processes on different factors (intensity of metabolism, oxygen concentration, temperature, etc.); and (iv) since the cell is not homogenous structure, RCS concentrations may differ to large extent in different cellular compartments. In addition, there are no standard techniques to measure RCS concentration *in vivo*, therefore controversial results can be obtained in different laboratories. Nonetheless, numerous studies report the RCS levels in biological samples. For instance, in plasma of healthy individuals the total concentration of RCS derived from lipid peroxidation is found below 1 *μ*M [10]. The physiological concentration of 4-HNE and MGO in plasma ranges from 0.3 to 0.7 *μ*M and from 0.12 to 0.65 *μ*M, respectively [8, 34, 70–72]. So, if the concentration of RCS does not exceed “normal” level, RCS involved in many of the cellular functions may have beneficial effects.

Phagocytic white blood cells that are of central importance in host defense mechanisms implicate RCS against invading pathogens. It is demonstrated that, besides certain ROS, myeloperoxidase generates such RCS as glycolaldehyde, 2-hydroxypropanal, acetone, and acrolein [52, 73]. Being highly reactive and toxic, some RCS are found to be potent anticancer agents. In the 1960s, it was proposed and then provided strong experimental evidence that MGO acted as an anticancer agent [72, 74–77]. Subsequent studies had indicated that MGO inhibited both glycolysis and mitochondrial respiration of specifically malignant cells [76, 78, 79]. Besides anticancer effect, RCS demonstrate antibacterial, antiprotozoal, antifungal, and antiviral activity [72].

## 5. Reactive Carbonyls in ****Signaling/Transcription Regulation

Understanding of the roles of RCS in intracellular signaling has evolved during the last decades. This was preceded by a discussion of RCS ability to participate in signaling/transcription regulation. The main question was how RCS meet the requirements for signaling molecules? Regardless of the nature of molecules, they can be recognized as signal if: (i) their level is tightly controlled *in vivo*; (ii) they are sufficiently stable, small, and hydrophobic to diffuse across biological membranes; (iii) they bind to specific receptors, triggering a chain of events within the cell; and (iv) their signaling effects are reversible. The enzymatic control of RCS production/elimination, RCS ability to cross biological membranes and diffuse for relatively long distances are the undoubted arguments for signaling role of reactive carbonyls. Recent studies from several laboratories show that RCS activate specific receptors [8, 11, 33]. It is also supposed that degradation and resynthesis of RCS-modified proteins are involved in the reversible aspect of RCS signaling [8]. For all the above-mentioned reasons, RCS seem among the best candidates for signaling purposes.

**Figure 5:**
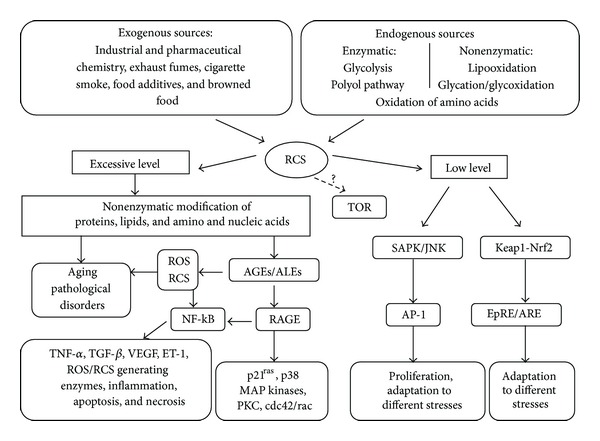
Involvement of reactive carbonyl species in signaling/transcription regulation.

Accumulating evidence from the last decades has shown that such RCS as 4-HNE, MDA, MGO, and GO can function as messengers that activate or inhibit signaling pathways under physiologic or pathologic conditions (Figure 5). They affect signaling mechanisms in a concentration- and time-dependent manner [10–13]. It has been shown, for example, that low levels of 4-HNE promote proliferation [80], but at higher concentrations it induces differentiation and apoptosis [81–83]. The underlying mechanisms by which RCS act as signaling messengers have been discussed extensively [8, 11, 80, 84–87]. Several cell signaling pathways, including the stress responses, proapoptotic events, kinase/phosphatase activities, and nuclear transcription factor function can be modulated by RCS in microorganisms, plant, and animals [85, 86, 88].

Numerous studies from different laboratories using a variety of mammalian cell lines have shown that 4-HNE induces SAPK/JNK signaling pathway [11, 81, 89, 90]. SAPK/JNK is stress-activated protein kinase/c-Jun NH(2)-terminal kinase, a member of MAPK family, activated by different types of stress and extracellular signals. SAPK/JNK activation plays essential role in organogenesis during mammal's development by regulating cell survival, apoptosis, and proliferation [91]. In hepatic cells, 4-HNE activates JNK through direct binding [89]. In other cells, 4-HNE activates JNK through the redox-sensitive MAPK kinase cascade [90]. It is suggested that 4-HNE-induced JNK activation promotes its translocation in the nucleus where JNK-dependent phosphorylation of c-Jun and the transcription factor activator protein (AP-1) binding take place [81, 92]. The AP-1 proteins are highly conserved among eukaryotes and belong to unspecific group of transcription factors controlling gene response to different signals. In mammalian cells, AP-1 proteins regulate the transcription of a number of genes involved in proliferation, differentiation, immune response, and adaptation to different stresses [88].

The vast majority of RCS, including 4-HNE and MDA, modulate transcription through the Keap1-Nrf2 pathway, which regulates the electrophile response element/antioxidant response element (EpRE/ARE) [33, 88, 93]. The activity of transcription factor Nrf2 (NF-E2-related factor 2) is dependent on its redox-sensitive inhibitor Keap1 (kelch-like ECH-associated protein 1). Under nonstressful conditions, the transcription factor Nrf2 is bound to Keap1. This complex promotes the ubiquitination of Nrf2 that followed by proteasomal degradation [93, 94]. Under cell exposure to RCS due to change of its conformation Keap1 becomes unable to form the complex with Nrf2 that results in the increased Nrf2 concentration. Further Nrf2 migrates into the nucleus, where it upregulates the transcription of target genes encoding superoxide dismutase, catalase, peroxiredoxin, glutathione peroxidase, thioredoxin reductase, *γ*-glutamylcysteine synthase, glutathione reductase thioredoxine reductase, heme oxygenase, quinone reductase, glutathione S-transferases, glutathione reductase, and other defensive proteins [12, 84, 87, 93, 95]. Interestingly, the *Arabidopsis thaliana* genome does not appear to encode Nrf2 homologues, although there are genes showing similarity to Keap1 that are considered to be involved in RCS signaling in plants [84]. The strong parallels in RCS stimulated gene expression are found in plants and animals (e.g., glutathione S-transferases, glutamylcysteine ligase, glutathione reductase, thioredoxin reductase, quinone reductase, heme oxygenase, and epoxide hydrolase). In *Saccharomyces cerevisiae*, some of these genes are under control of the yeast AP-1, called Yap1p transciptional factor that can be activated by MGO [96].

In the middle of the 1980s, it was demonstrated that macrophages could specifically recognize, uptake, and degrade AGEs/ALEs-modified proteins *in vitro *[97]. This observation led to an active search for high affinity AGEs/ALEs receptors on various cells. The first discovered multiligand receptor able to bind AGEs/ALEs-modified proteins with high affinity was RAGE (the receptor for AGE, member of the immunoglobulin superfamily of cell surface molecules) [98]. RAGE interacts with distinct molecules implicated in homeostasis, inflammation, and certain diseases [55, 99].

In the presence of extracellular AGEs/ALEs, susceptible cells can rapidly upregulate expression of RAGE on their membranes (Figure 5). Engagement of RAGE by a ligand triggers activation of key cell signalling pathways such as p21^ras^, protein kinase C, MAP kinases, cdc42/rac, and NF-kB, thereby reprogramming cellular properties [2, 99, 100]. For example, activation of nuclear factor NF-kB due to AGEs/ALEs and RAGE interaction was shown to be involved in the regulation of the gene transcription for various factors: endothelin-1 (ET-1), vascular endothelial growth factor (VEGF), transforming growth factor *β* (TGF-*β*), and tumor necrosis factor *α* (TNF-*α*) [55, 101]. Also, NF-kB controls the expression of almost 100 proinflammatory genes encoding cytokines, adhesion molecules, and ROS/RCS generating enzymes such as NADPH-oxidase, superoxide dismutase, inducible nitric oxide synthase, and myeloperoxidase [100–103].

Search for new AGEs/ALEs receptors has resulted in the identification of macrophage scavenger receptors (MSR) types A and B1 (CD36), oligosaccharyl transferase-48 termed AGE receptor 1 (AGE-R1), 80K-H phosphoprotein (AGE-R2), and galectin-3 (AGE-R3) [100, 101], but the best studied is the RAGE receptor.

The TOR (target of rapamycin) signaling pathway integrates a large number of environmental changes and regulates cell growth and aging through control of certain anabolic and catabolic processes [104]. In clinical biology, TOR is implicated in many diseases. Although there is no information on the relationship between RCS and TOR pathway, it has been suggested that rapamycin decreases MGO generation *in vivo* by inhibiting TOR activity [105]. In our preliminar experiments, yeast parental strain and its isogenic derivatives defective in TOR demonstrated significantly different intracellular levels of RCS and susceptibilities to RCS-induced stress. Therefore, potential interplay between certain reactive carbonyls and TOR signaling cascade cannot be excluded.

## 6. Conclusions

There is sufficient experimental evidence that reactive species, and RCS in particular, have the ability to modulate homeostasis at various levels, probably by both damaging biological molecules and participating in signaling/transcription regulation. Different signaling networks are involved in both deleterious and beneficial effects of reactive carbonyls. This dual nature of RCS biological effects appears to be dose- and time-dependent. Since RCS can modulate such biological processes as proliferation, differentiation, reproduction, maintenance of metabolic equilibrium, immune response, adaptation to different stresses, apoptosis, necrosis, aging and development of certain pathologies, new tools are required to decipher the mechanisms underlying the dual effects. Understanding of the relationship between metabolism of RCS and the development of pathological disorders and diseases will also make a contribution not only to our knowing of how RCS cause biological effects, but also on how to define effective therapeutic approaches to prevent them.

## Figures and Tables

**Table 1:** Carbonyl compounds and sources of their generation *in vivo*.

Nonenzymatic			Enzymatic	
Peroxidation of lipids	Glycation	Oxidation of amino acids	Polyol pathway	Glycolysis
Malondialdehyde 4-Hydroxy-trans-2-nonenal 4-Oxo-trans-2-nonenal Glyoxal Methylglyoxal Acrolein Crotonaldehyde Hexanal	Glyoxal Methylglyoxal Glucosone 3-Deoxyglucosone Acrolein	Glyoxal Methylglyoxal Acrolein Glycolaldehyde 2-Hydroxypropanal	3-Deoxyglucosone 3-Deoxyfructose	Acetaldehyde Glyceraldehyde-3-phosphate Dioxyacetone phosphate Methylglyoxal

## Abbreviations

AP-1: Activator protein 1
3-DG: 3-deoxyglucosone
4-HNE: 4-hydroxy-2-nonenal
AGEs: Advanced glycation end products
ALEs: Advanced lipoxidation end products
EpRE/ARE: Electrophile response element/antioxidant response element
GO: Glyoxal
Keap1: Kelch-like ECH-associated protein 1
MDA: Malondialdehyde
MGO: Methylglyoxal
Nrf2: NF-E2-related factor 2
RAGE: Receptor for AGE; RCS, reactive carbonyl species
ROS: Reactive oxygen species
SAPK/JNK: Stress-activated protein kinase/c-Jun NH(2)-terminal kinase.
